# Investigating the relationship between DNA methylation age acceleration and risk factors for Alzheimer's disease

**DOI:** 10.1016/j.dadm.2018.05.006

**Published:** 2018-06-21

**Authors:** Daniel L. McCartney, Anna J. Stevenson, Rosie M. Walker, Jude Gibson, Stewart W. Morris, Archie Campbell, Alison D. Murray, Heather C. Whalley, David J. Porteous, Andrew M. McIntosh, Kathryn L. Evans, Ian J. Deary, Riccardo E. Marioni

**Affiliations:** a Medical Genetics Section, Centre for Genomic and Experimental Medicine, Institute of Genetics and Molecular Medicine, University of Edinburgh, Edinburgh, Scotland; b Division of Psychiatry, University of Edinburgh, Royal Edinburgh Hospital, Edinburgh, Scotland; c Aberdeen Biomedical Imaging Centre, University of Aberdeen, Aberdeen, Scotland; d Centre for Cognitive Ageing and Cognitive Epidemiology, University of Edinburgh, Edinburgh, Scotland; e Department of Psychology, University of Edinburgh, Edinburgh, Scotland

**Keywords:** DNA methylation, Epigenetic clock, Alzheimer's disease, Genetics, Generation Scotland

## Abstract

**Introduction:**

The “epigenetic clock” is a DNA methylation–based estimate of biological age and is correlated with chronological age—the greatest risk factor for Alzheimer's disease (AD). Genetic and environmental risk factors exist for AD, several of which are potentially modifiable. In this study, we assess the relationship between the epigenetic clock and AD risk factors.

**Methods:**

Multilevel models were used to assess the relationship between age acceleration (the residual of biological age regressed onto chronological age) and AD risk factors relating to cognitive reserve, lifestyle, disease, and genetics in the Generation Scotland study (n = 5100).

**Results:**

We report significant associations between age acceleration and body mass index, total cholesterol to high-density lipoprotein cholesterol ratios, socioeconomic status, high blood pressure, and smoking behavior (Bonferroni-adjusted *P* < .05).

**Discussion:**

Associations are present between environmental risk factors for AD and age acceleration. Measures to modify such risk factors might improve the risk profile for AD and the rate of biological ageing. Future longitudinal analyses are therefore warranted.

## 1 Introduction

DNA methylation is an epigenetic modification typically characterized by the addition of a methyl group to a cytosine-guanine dinucleotide. Both genetic and environmental factors influence DNA methylation, which in turn can regulate gene expression [1]. The “epigenetic clock” is an estimation of biological age derived from DNA methylation data and is strongly correlated with chronological age [2]. From biological age, a measure of age acceleration can be obtained based on the difference between an individual's biological (estimated) and chronological (actual) age. Age acceleration has been linked to a range of age-related health outcomes, including increased Alzheimer's disease (AD) pathology [3], reduced cognitive and physical fitness [4], and an increase in all-cause mortality [5]. The epigenetic clock has therefore been proposed as a biomarker of ageing and may be predictive of age-related disorders, such as dementia [6].

Dementia is one of the leading global health concerns of the 21st century. The most common form of dementia is AD. Lifestyle factors such as smoking have been linked to an increased risk of AD [7], as have disease-related factors including type 2 diabetes (T2D) and high blood pressure (HBP) [8], [9]. Moreover, resilience to age-related brain changes (e.g., cognitive reserve) has been linked to AD risk [10]. Factors such as educational attainment and socioeconomic status have been proposed as proxy measures of cognitive reserve, and lower levels of these are established AD risk factors [11], [12]. Genetic studies of AD have revealed several risk factors [13], with the *APOE* locus (encoding apolipoprotein E) being among the strongest [14].

A recent review [15] suggested that up to a third of cases of all-cause dementia might be delayed by actively addressing its modifiable risk factors. The present study aims to investigate the relationship between epigenetic age acceleration and both genetic and potentially modifiable environmental AD risk factors. Two measures of age acceleration were assessed in over 5000 individuals from the Generation Scotland cohort, intrinsic epigenetic age acceleration (IEAA) and extrinsic epigenetic age acceleration (EEAA). These measures are described in greater detail in the methods section. Briefly, IEAA is a measure of age acceleration that is independent of age-related changes in the cellular composition of blood [16], whereas EEAA captures the age-related functional decline of the immune system. Age is the strongest risk factor for AD [17], and epigenetic age is a robust predictor of chronological age. We therefore hypothesize that individuals with poorer profiles for AD risk factors display accelerated ageing in comparison to those with more favorable profiles.

## 2 Methods

### 2.1 The Generation Scotland cohort

Details of the Generation Scotland: Scottish Family Health Study (GS:SFHS) have been described previously [18], [19]. Briefly, the cohort comprises 23,960 individuals, each with at least one family member participating in the study. DNA samples were collected for genotype and DNA methylation profiling along with detailed clinical, lifestyle, and sociodemographic data. The present study comprised 5200 individuals from the cohort for whom DNA methylation data were available. A summary of all variables assessed in this analysis is presented in Table 1.

**Table 1:** Summary of variables assessed in the Generation Scotland cohort

Variable	N	Mean	SD
Chronological age (years)	5100	48.51	13.99
Horvath's estimated age (years)	5100	52.60	11.59
Hannum's estimated age (years)	5100	39.42	11.68
Body mass index (BMI; kg/m^2^)	4977	27.03	5.37
Smoking (pack years)∗	4997	9.13	17.28
High-density lipoprotein (HDL) cholesterol (mmol/L)	4948	1.49	0.42
Total cholesterol (mmol/L)	4960	5.13	1.09
Total:HDL cholesterol (ratio)	4948	3.67	1.22
	N	Mean	IQR

Socioeconomic status (SIMD, rank)	4728	4230	2148.5–5423
Education†	4816	4	3–6
AD polygenic risk score	4994	1.7 × 10^−4^	1.6 × 10^−4^ to 1.9 × 10^−4^
Sex (male/female)	1918/3083	-	-
Type 2 diabetes (yes/no)	171/4830	-	-
High blood pressure (yes/no)	768/4830	-	-
AD family history (yes/no)	834/4167	-	-
*APOE* (ε2ε2)	27	-	-
*APOE* (ε2ε3)	572	-	-
*APOE* (ε2ε4)	108	-	-
*APOE* (ε3ε3)	2952	-	-
*APOE* (ε3ε4)	1126	-	-
*APOE* (ε4ε4)	124	-	-

Abbreviations: AD, Alzheimer's disease; BMI, body mass index; IQR, interquartile range; SIMD, Scottish Index of Multiple Deprivation.

∗ The following smoking categories were available: current smoker (N = 939); former smoker, stopped within past 12 months (N = 158); former smoker, stopped more than 12 months ago (N = 1309); never smoker (N = 2533). Data were unavailable for 62 participants.

† Education was measured as an ordinal variable—0: 0 years; 1: 1–4 years; 2: 5–9 years; 3: 10–11 years; 4: 12–13 years; 5: 14–15 years; 6: 16–17 years; 7: 18–19 years; 8: 20–21 years; 9: 22–23 years; and 10: ≥24 years.

### 2.2 Ethics

All components of GS:SFHS received ethical approval from the NHS Tayside Committee on Medical Research Ethics (REC reference number: 05/S1401/89). GS:SFHS has also been granted research tissue bank status by the NHS Tayside Committee on Medical Research Ethics (REC reference number: 10/S1402/20), providing generic ethical approval for a wide range of uses within medical research.

### 2.3 GS:SHFS DNA methylation

Genome-wide DNA methylation was profiled in blood samples from 5200 individuals using the Illumina HumanMethylationEPIC BeadChips. Quality control was conducted using R [20]. ShinyMethyl [21] was used to plot the log median intensity of methylated versus unmethylated signal per array with outliers being excluded upon visual inspection. The software package WateRmelon [22] was used to remove (1) samples in which ≥1% of cytosine-guanine dinucleotides had a detection *P* value in excess of .05; (2) probes with a beadcount of less than 3 in more than 5 samples; and (3) probes in which ≥0.5% of samples had a detection *P* value in excess of .05. ShinyMethyl was used to exclude samples in which predicted sex did not match recorded sex. This left a sample of 5101 available for analysis.

### 2.4 Calculation of age acceleration

Methylation-based estimates of age were calculated using the online age calculator (https://dnamage.genetics.ucla.edu/) developed by Horvath [23]. Normalized GS:SHFS DNA methylation data were used as input for the algorithm, and data underwent a further round of normalization by the age calculator. Two measures of age acceleration were calculated, IEAA and EEAA. IEAA is defined as the residual term of a multivariate model regressing estimated Horvath methylation age [23] on chronological age, fitting counts of naive CD8+ T-cells, exhausted CD8+ T-cells, plasmablasts, CD4+ T-cells, natural killer cells, monocytes, and granulocytes estimated from the methylation data. IEAA therefore does not consider age-related changes in the cellular composition of blood. Horvath's measure of methylation age is also consistent across multiple tissue types, including brain [23]. Conversely, the estimate of EEAA tracks age-related changes in blood cell composition as well as intrinsic epigenetic changes. EEAA is calculated first by calculating a weighted average of Hannum's DNA methylation age [24] and three cell types whose abundance is known to change with age (naive cytotoxic T-cells, exhausted cytotoxic T-cells, and plasmablasts) using the approach described by Klemera and Doubal [25]. EEAA is defined as the residual term of a univariate model regressing the weighted estimated age on chronological age. EEAA correlates with age-related changes in the cellular composition of blood, thereby capturing a degree of immunosenescence.

### 2.5 Definition of AD risk factors

AD risk factors were divided into four categories: (1) cognitive reserve, (2) disease, (3) lifestyle, and (4) genetics. Cognitive reserve factors comprised education years and socioeconomic status as measured by the Scottish Index of Multiple Deprivation (SIMD). Education was measured as an ordinal variable—0: 0 years; 1: 1–4 years; 2: 5–9 years; 3: 10–11 years; 4: 12–13 years; 5: 14–15 years; 6: 16–17 years; 7: 18–19 years; 8: 20–21 years; 9: 22–23 years; and 10: ≥24 years. The SIMD is comprised of ranks for data zones throughout Scotland from 1 (most deprived) to 6505 (least deprived). For each data zone, ranks are calculated based on income, employment, health, education, skills and training, housing, geographic access, and crime. Disease-related factors comprised self-reported type 2 diabetes status and HBP status. Lifestyle factors comprised smoking pack years (defined as packs smoked per day times years as a smoker), body mass index (BMI), high-density lipoprotein (HDL), total cholesterol, and total:HDL cholesterol ratio. Genetic factors comprised family history (defined as having a parent or grandparent with AD), AD polygenic risk score (PGRS), and *APOE* ε4 carrier status.

### 2.6 Calculation of AD PGRS

PGRS for AD was created for all individuals with genotype data in the GS:SHFS cohort. All autosomal SNPs which passed quality control were included in the calculation of the PGRS for AD (see Supplementary Information for quality control parameters). PGRS for AD was estimated using summary statistics from an independent GWAS of AD (17,008 cases; 37,154 controls) conducted by the International Genomics of Alzheimer's Project [13]. PGRS was estimated using the PRSice software package, according to previously described protocols [26], with LD threshold and distance threshold for clumping of R^2^ > 0.25 and 250 kb, respectively. After excluding SNPs within a 500-kb region of *APOE*, a score was created for each individual, using all possible remaining SNPs, in accordance with previous GS:SFHS analyses [27].

### 2.7 Statistical analysis

Multilevel models were built in R [20], assessing the relationship between epigenetic age acceleration (IEAA and EEAA) and factors related to cognitive reserve, disease, lifestyle, and genetics. In each model, the AD risk factor was fitted as the outcome; chronological age, sex, and age acceleration were fitted as fixed effects; and pedigree information was fitted as a random effect to control for genetic relatedness within the cohort. Models were built using the *MCMCglmm()* function from the MCMCglmm R package [28]. Correction for multiple testing was applied separately to IEAA- and EEAA-based analyses using the Bonferroni method. Numeric variables were scaled to have zero mean and unit variance.

## 3 Results

### 3.1 Estimation of epigenetic age

Methylation data from 5101 individuals were submitted to the online age calculator. One individual was flagged for an incorrect sex prediction and was omitted from downstream analysis, leaving 5100 individuals. A summary of chronological and estimated ages in the GS:SHFS cohort is provided in Table 1. Both Horvath's and Hannum's estimates of biological age were strongly correlated with chronological age (r = 0.94 and 0.93, respectively). As reported previously [29], there was a strong effect of biological sex on age acceleration, with men showing greater acceleration than women (mean EEAA: males = 0.47 and females = −0.3 years, *P* = 3.58 × 10^−12^; Mean IEAA: males = 1.13 and females = −0.71 years, *P* = 8.68 × 10^−53^).

### 3.2 Cognitive reserve and epigenetic age acceleration

**Fig. 1:**
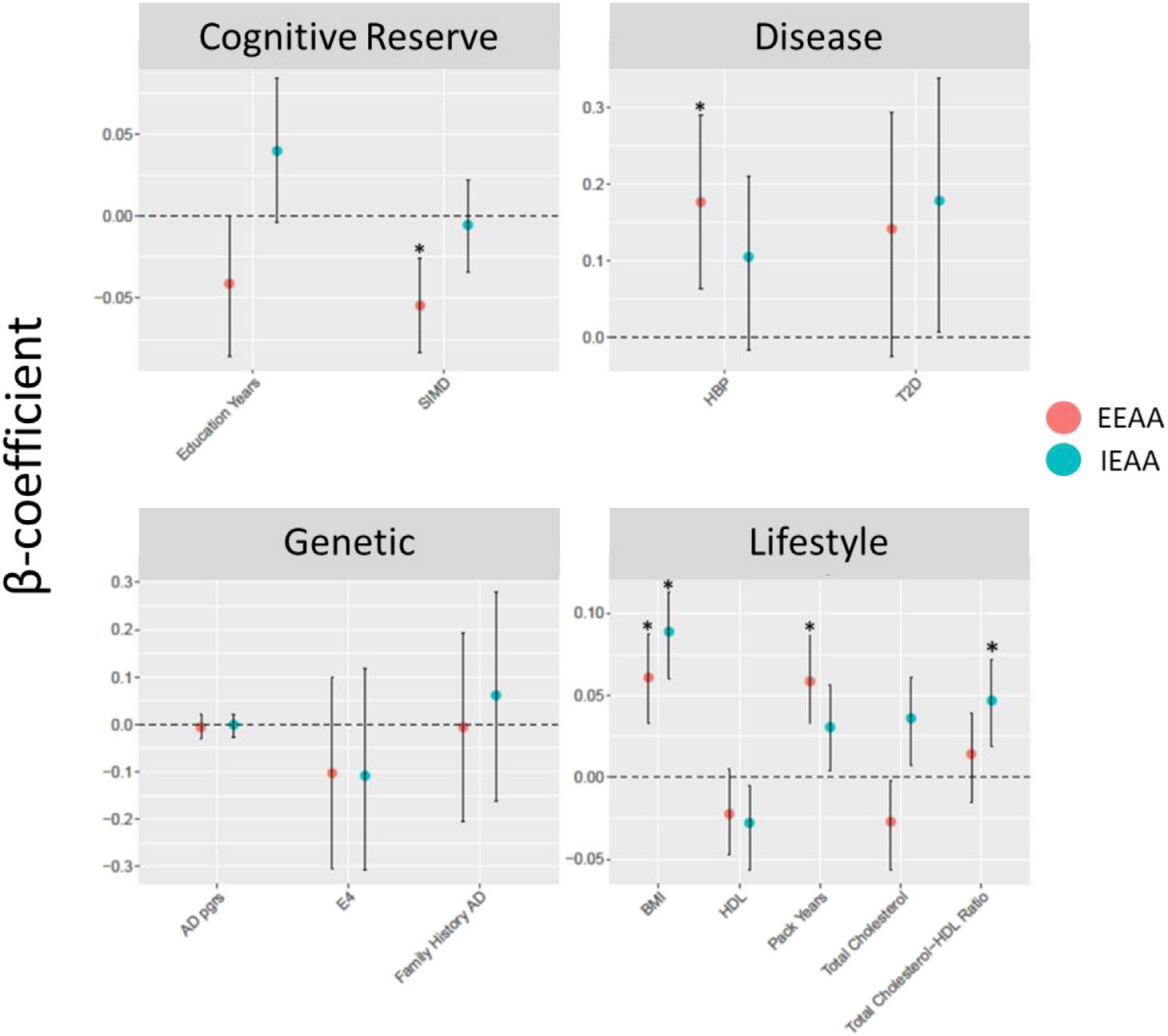
Effects of AD risk factors on age acceleration. Plots are separated into four groups of AD risk factors: cognitive reserve, disease, lifestyle, and genetic. Standardized model β coefficients (i.e., effect sizes) are presented along the y-axes, whereas risk factors are presented along the x-axes. Points are colored by EEAA (red) and IEAA (blue). Error bars show the 95% CI. Points accompanied by an asterisk (*) represent measures significantly associated with age acceleration at a Bonferroni *P* < .05. Abbreviations: 95% CI, 95% confidence interval; AD, Alzheimer’s disease; BMI, body mass index; EEAA, extrinsic epigenetic age acceleration; HBP, high blood pressure; HDL, high-density lipoprotein cholesterol; IEAA, intrinsic epigenetic age acceleration; PGRS, polygenic risk score; SIMD, Scottish Index of Multiple Deprivation; T2D, type 2 diabetes. Effect sizes represent SD increase/decrease in epigenetic age per 1 SD increase/decrease in risk factor (disease positive for HBP and T2D, positive *APOE* ε4 carrier status, and positive family history of AD).

Two cognitive reserve factors were evaluated for association with age acceleration: socioeconomic status based on the SIMD and education years (Table 2; Fig. 1). No significant associations were present between these factors and IEAA. Nominally significant negative associations (at *P* < .05) were observed between EEAA and both education and SIMD (0.076 standard deviation [SD] decrease in EEAA per SD increase in education, *P* = .048; 0.05 SD decrease in EEAA per SD increase in SIMD, *P* < .001).

**Table 2:** Age acceleration and AD risk factors

Risk factor	IEAA			EEAA		
	β	95% CI	*P*	β	95% CI	*P*
Cognitive reserve						
Socioeconomic status (SIMD, SD)	−0.005	−0.034 to 0.022	.71	−0.056	−0.083 to −0.026	**<.001**
Education∗ (per unit)	0.0398	−0.0037 to 0.084	.09	−0.041	−0.085 to 0.0004	.058
Disease						
Type 2 diabetes (yes/no)	0.178	0.007−0.338	.06	0.142	−0.024 to 0.294	.08
High blood pressure (yes/no)	0.105	−0.016 to 0.21	.078	0.177	0.064–0.29	**.002**
Lifestyle						
Body mass index (BMI; kg/m^2^)	0.089	0.06–0.11	**<.001**	0.061	0.03–0.087	**<.001**
Smoking† (pack years)	0.031	0.004–0.06	.028	0.059	0.0325−0.086	**<.001**
High-density lipoprotein (HDL) cholesterol (mmol/L)	−0.028	−0.056 to −0.0053	.032	−0.022	−0.047 to 0.005	.098
Total cholesterol (mmol/L)	0.036	0.007−0.06	.004	−0.027	−0.056 to −0.002	.056
Total:HDL cholesterol (ratio)	0.047	0.019−0.072	**<.001**	0.014	−0.015 to 0.039	.33
Genetic						
AD polygenic risk score (SD)	−0.002	−0.026 to 0.022	.896	−0.007	−0.03 to 0.02	.6
AD family history (yes/no)	0.06	−0.162 to 0.279	.614	−0.007	−0.21 to 0.19	.94
*APOE* (ε4 carrier)	−0.107	−0.307 to 0.119	.308	−0.103	−0.306 to 0.1	.3

Abbreviations: AD, Alzheimer's disease; BMI, body mass index; CI, confidence interval; EEAA, extrinsic epigenetic age acceleration; IEAA, intrinsic epigenetic age acceleration; SIMD, Scottish Index of Multiple Deprivation.

NOTE. Significant associations after accounting for multiple comparisons are highlighted in bold (*P* < .004).

∗ Education was measured as an ordinal variable. 0: 0 years; 1: 1–4 years; 2: 5–9 years; 3: 10–11 years; 4: 12–13 years; 5: 14–15 years; 6: 16–17 years; 7: 18–19 years, 8: 20–21 years; 9: 22–23 years; and 10: ≥24 years.

† The following smoking categories were available: current smoker (N = 939); former smoker, stopped within past 12 months (N = 158); former smoker, stopped more than 12 months ago (N = 1309); never smoker (N = 2533), and not coded (N = 62).

### 3.3 Disease-related risk factors and epigenetic age acceleration

We assessed the relationship between age acceleration and two disease-related risk factors such as T2D and HBP (Table 2; Fig. 1). No significant associations were observed between either measure of epigenetic age acceleration and T2D. There was a significant relationship between extrinsic age acceleration and HBP (*P* = .002; 0.177 SD increase in EEAA for individuals with HBP).

### 3.4 Lifestyle-related risk factors and epigenetic age acceleration

Four factors related to lifestyle were considered: (1) BMI, (2) smoking habits (pack years), (3) HDL, and (4) total cholesterol (Table 2; Fig. 1). Higher values of both measures of epigenetic age acceleration were observed with higher BMI (IEAA: 0.089 SD increase per SD increase in BMI, *P* < .001; EEAA: 0.061 SD increase per SD increase of BMI, *P* < .001) and more pack years (IEAA: 0.031 SD increase per SD increase in smoking pack years, *P* = .028; EEAA: 0.059 SD increase per SD increase in smoking pack years, *P* < .001). Greater IEAA was associated with lower levels of HDL cholesterol (0.028 SD decrease in IEAA per SD increase of mmol/L HDL, *P* = .032) and higher levels of total cholesterol (0.036 SD increase in IEAA per SD increase of mmol/L total cholesterol, *P* = .004). A significant positive association was present between IEAA and total:HDL cholesterol ratios (0.047 SD increase in IEAA per SD increase in ratio of total:HDL cholesterol, *P* < .001). There were no significant associations observed between EEAA and any of the three cholesterol-related metrics assessed.

### 3.5 Genetic risk factors and epigenetic age acceleration

Three genetic risk factors for AD were assessed for association with age acceleration: (1) family history, (2) AD PGRS, and (3) *APOE* ε4 carrier status (Table 2; Fig. 1). No significant associations were present between any of the genetic risk factors assessed and either measure of epigenetic age acceleration.

### 3.6 Correction for multiple testing

Applying a Bonferroni correction separately for the IEAA and EEAA regressions (0.05/12 = adjusted *P* < .0042) identified significant IEAA associations with BMI and total:HDL cholesterol ratio (BMI adjusted *P* < 0.001; total:HDL cholesterol ratio adjusted *P* < .001) and significant EEAA associations with SIMD, BMI, HBP status, and smoking (SIMD adjusted *P* < .001; BMI adjusted *P* < .001; HBP adjusted *P* = .002; and smoking adjusted *P* < .001). Of these, increased age acceleration was associated with increased total:HDL cholesterol ratios, BMI, smoking levels, social deprivation, and HBP status.

## 4 Discussion

In the present study, we hypothesized that age acceleration might be associated with AD risk factors in the Generation Scotland cohort. Using both intrinsic (cell-adjusted) and extrinsic (immune system–associated) estimates of epigenetic age acceleration in a cohort of 5100 individuals, we identified significant associations between multiple AD risk factors and age acceleration. Several of the AD risk factors associated with age acceleration are potentially modifiable lifestyle factors, suggesting the rate of epigenetic ageing can be altered through behavioral changes.

Biological age has been linked to an increased risk of all-cause mortality and is strongly correlated with chronological age [5]. The epigenetic clock has been proposed as a biomarker of ageing as well as a predictor of an individual's health and susceptibility to age-related health outcomes [3], [5]. As chronological age increases, so does the risk of dementia. Individuals with greater age acceleration (i.e., with greater epigenetic age relative to chronological age) have slightly poorer cognitive ability [4] and a modest increase in burden of pathological hallmarks of dementia [3].

Of the risk factors assessed, BMI and smoking levels were associated (at a nominal significance threshold) with both estimates of age acceleration. BMI has previously been associated with an increased risk of dementia and AD when it is high in middle age and low in old age [30], [31]. Consistent with our findings, others have observed an association between higher BMI and increased age acceleration using both Hannum- and Horvath-based algorithms [23], [24], [32]. Previous studies have failed to find associations between smoking levels and epigenetic age acceleration [16], [33]. Our findings of a significant positive association between self-reported smoking and both measures of age acceleration may be attributable to our larger sample size (N = 4997 individuals compared with maximum N = 978 individuals with smoking data available [33]), although only EEAA was significantly associated with smoking after correction for multiple testing.

In the present study, factors relating to cholesterol were associated with age acceleration based on the intrinsic (cell-adjusted) estimate of epigenetic age acceleration (at a nominal significance threshold for HDL cholesterol and total cholesterol). HDL levels were negatively correlated with epigenetic age acceleration, whereas both total cholesterol levels and total:HDL cholesterol ratio were positively correlated with age acceleration. To our knowledge, significant associations between methylation-based estimates of age acceleration and total:HDL cholesterol ratios have not been reported to date. Consistent with our findings, others have observed an association between lower HDL cholesterol and increased age acceleration [32]. A relationship between increased age acceleration and both total and HDL cholesterol levels using a transcriptomic estimate of biological age has also been reported [34]. HDL cholesterol, colloquially known as “good cholesterol,” primarily functions in lipid transport. Higher levels of HDL cholesterol have been linked to a reduction in cardiovascular disease risk [35] as well as a decreased risk of AD and dementia [36], [37]. Conflicting evidence exists for the association between mid-life levels of total cholesterol and dementia risk [38], [39]; however, studies have consistently reported an inverse association between total cholesterol levels and AD risk in elderly individuals [40], [41], [42]. Longitudinal analyses have revealed different trajectories of BMI in dementia cases compared with controls [31]. Similarly, longitudinal analyses have also indicated that mid- to late-life trajectories of cholesterol levels are related to both *APOE* genotype [43] and dementia status [44]. *APOE*, a strong genetic risk factor for AD, also functions in lipid transport. The association between cholesterol levels and AD risk, coupled with the functions of *APOE* and other genetic risk factors (e.g., *SORL1*) [13], supports the role of lipid metabolism and transport in dementia [45], [46].

For the proxy measures of cognitive reserve, both educational attainment and socioeconomic status were associated with EEAA. Of the two, however, only socioeconomic status remained significant after Bonferroni correction. Those with fewer education years showed increased age acceleration, as did individuals from more deprived socioeconomic backgrounds. Individuals with increased levels of education have displayed delays in the age of onset of dementia [47]. Lower levels of education are also associated with an increased risk of transitioning from a cognitively normal to a cognitively impaired state [48]. Consistent with our findings, others have reported a similar pattern between EEAA and educational attainment [32], [49]. Moreover, an inverse relationship has previously been reported between socioeconomic status and a measure of age acceleration also based on the algorithm by Hannum et al. [24], [50]. The manifestation of biological differences linked to social deprivation is possibly due to the association between socioeconomic status and other, more biologically direct, risk factors for dementia. For example, several lifestyle-related AD risk factors have been shown to be associated with socioeconomic status, including smoking and BMI [51], [52].

Of the disease-related AD risk factors, there were no associations between T2D and either measure of age acceleration. However, a significant association was observed between HBP status and the extrinsic estimate of age acceleration. Hypertension is prevalent among older individuals, and its link with dementia is well established [53], [54]. Consistent with our findings, others have reported an association between blood pressure and EEAA [32]. It should be noted, however, that the study cohort was limited to postmenopausal women.

No significant associations were observed between either measure of age acceleration and any of the genetic risk factors assessed. Epigenetic age acceleration effects of environmental factors such as smoking and cholesterol may be more visible in blood because of direct contact with the tissue. Although genetic risk factors should be consistent across all tissues, it is possible that they only influence epigenetic age acceleration in cell types in which AD pathology is primarily observed (i.e., brain tissue).

After Bonferroni correction, only BMI, cholesterol ratios, smoking, HBP status, and socioeconomic status were associated with age acceleration. With the exception of socioeconomic status, all are traits that can directly impact on cardiometabolic health. This highlights a well-established overlap between AD risk and that of cardiovascular disease [54].

With a sample size in excess of 5000 individuals, this is among the largest single-cohort studies of DNA methylation–based ageing to date. Recent analyses of DNA methylation–based ageing have been undertaken with sample sizes of a similar scale, using multiple cohorts [50], [55]. An advantage of the present study is that the cohort is derived from a single population. This minimizes the likelihood of confounders such as genetic and phenotypic stratification, which might be observed in studies comprising multiple populations. Moreover, the use of a comprehensively genotyped and phenotyped cohort has permitted the assessment of both genetic and environmental AD risk factors and their relationship with epigenetic ageing. This resource is further strengthened by the potential for data linkage to medical records and recontact of participants, making future longitudinal analyses possible. The cross-sectional design of the present study poses a limitation as it does not permit the assessment of longitudinal changes in age acceleration in response to altered lifestyle habits. However, such a study might be informative in determining whether the trajectory of biological age can be modified through efforts to reduce the risk of AD and other forms of dementia. An additional limitation relates to the absence of AD biomarker data in the Generation Scotland cohort. Deriving high-quality measurements for biomarkers such as tau and amyloid-β in live subjects can be invasive and costly. However, the recent development of high-performance assays for plasma-based tau and amyloid-β may provide a noninvasive, cost-effective alternative for future analyses [56], [57]. With the exception of BMI, significant associations were specific to either IEAA or EEAA. This discordance is possibly due to differences in the two estimates of age acceleration. As described in the methods section, IEAA does not reflect differences in blood cell composition that may be due to age while these differences are incorporated into the estimate of EEAA. HBP, smoking and socioeconomic status were associated with EEAA, but not IEAA. This may reflect a relationship between these risk factors and immunosenescence. There were no available measurements for immunosenesence-related markers in the Generation Scotland cohort. However, others have reported inverse associations between socioeconomic factors and interleukin-6 and C-reactive protein—two markers of immunosenescence [58], [59]. Moreover, several studies have reported an association between hypertension and elevated levels of C-reactive protein [60]. In contrast, total:HDL cholesterol ratio was associated with IEAA but not EEAA, possibly reflecting a relationship between this factor and “pure” epigenetic ageing (i.e., tissue-agnostic ageing).

In conclusion, we reported associations between both intrinsic and extrinsic measures of epigenetic age acceleration and environmental AD risk factors. However, no associations were present for the genetic risk factors assessed. At a nominal (*P* < .05) significance threshold, IEAA was associated with all of the lifestyle-related factors assessed, whereas EEAA was associated with HBP, BMI, smoking, and both cognitive reserve factors assessed. After Bonferroni correction, BMI, cholesterol ratios, smoking, HBP, and socioeconomic status remained significantly associated with epigenetic age acceleration. These have all been linked to cardiovascular disease risk, as well as AD risk [15], [61]. Risk factors such as cholesterol levels, smoking, blood pressure, and BMI can be modulated by behavioral changes with regard to exercise, dietary intake, and smoking behavior. The epigenetic clock is a robust predictor of chronological age, and the greatest risk factor for AD is advanced age [17]. Individuals displaying accelerated ageing have demonstrated increased AD neuropathology and lower cognitive test scores [3], [4]. In the present study, we observed a relationship between age acceleration and AD risk factors. It is reasonable to suggest that by improving one's AD risk profile where possible, the biological ageing process could be “slowed.”

**Research in Context**

1. Systematic review: Previous studies have identified a relationship between epigenetic ageing and age-related outcomes, including Alzheimer's disease pathology and reduced cognitive performance. In this study, we present the largest study of DNA methylation–based ageing to date. We assessed the relationship between the epigenetic clock and both genetic and environmental Alzheimer's disease risk factors in a cohort of over 5000 individuals.
2. Interpretation: We identified significant associations between epigenetic age acceleration and lifestyle-related risk factors, but not genetic risk factors. Potentially modifiable factors were including body mass index, total:high-density lipoprotein cholesterol ratios, smoking, high blood pressure, and socioeconomic status.
3. Future directions: We hypothesize that behavioral changes with an aim to modify such risk factors may improve individual risk profiles for Alzheimer's disease, potentially decreasing the rate of epigenetic age acceleration. As this hypothesis cannot be addressed due to the cross-sectional design of the present study, longitudinal analyses are warranted.
