# Supplementary material for: Investigating the relationship between DNA methylation age acceleration and risk factors for Alzheimer's disease
Source: Alzheimers Dement (Amst). 2018 Jun 21;10:429–37. doi: 10.1016/j.dadm.2018.05.006 (PMC6111045; doi:10.1016/j.dadm.2018.05.006)
Supplement: Supplementary Information [file mmc1.docx]

**Supplementary Information**

**Genotyping and quality control in the GS:SFHS cohort**

Blood and saliva samples were collected, following standard operating procedures, and stored at the Wellcome Trust Clinical Research Facility Genetics Core, Edinburgh, UK ([www.wtcrf.ed.ac.uk](http://www.wtcrf.ed.ac.uk/)), where DNA extraction and genotyping were carried out; details of sample collection and DNA extraction are provided elsewhere [1]. Samples were genotyped using the Illumina HumanOmniExpressExome-8v1.0 Beadchip and Infinium chemistry^2^ and processed using the Illumina GenomeStudio Analysis software v2011.1 (Illumina, San Diego, CA, USA). Quality control procedures on the raw genotypes removed individuals with an overall genotyping rate of less than 99%, SNPs with a minor allele frequency less than 1% or call rate under 99%, or a significant deviation from Hardy-Weinberg equilibrium (P≥1x10^-6^).

**References**

1. Smith BH, Campbell H, Blackwood D, Connell J, Connor M, Deary IJ, et al. Generation Scotland: The Scottish Family Health Study; a new resource for researching genes and heritability. BMC Med Genet 2006;7. doi:10.1186/1471-2350-7-74.
